# In Vivo Efficacy Study Showing Comparative Advantage of Bacterial Infection Prevention with Zip-type Skin Closure Device vs. Subcuticular Sutures

**DOI:** 10.7759/cureus.3102

**Published:** 2018-08-04

**Authors:** Bauback Safa, Amir Belson, Carol Meschter, Michelle Kelley, Daren Stewart, Kei Ichiryu, Sandra Biroc, Eric Storne

**Affiliations:** 1 The Buncke Clinic, CPMC, San Francisco, USA; 2 ZipLine Medical, Inc., Campbell, USA; 3 Comparative Biosciences, Inc., Sunnyvale, USA

**Keywords:** wound, closure, shear, dehiscence, scar, infection, bacteria, suture, ssi, hai

## Abstract

There remains a lack of understanding of how wound closure methods perform comparatively when exposed to patient-induced movement during healing and how they may contribute to bacterial infiltration in the wound site. The present study attempts to objectively quantify this gap. The study evaluates bacterial penetration and subsequent symptoms of infection of traditional sutures and an emerging tape-based, zip-type wound closure technology under physiologically relevant loading. In an in vivo model to simulate real-world conditions, the latter demonstrates better performance compared to commonly used sutures, holding the wound intact and minimizing bacterial penetration when subjected to simulated patient movement-induced stress.

## Introduction

Surgical site infections (SSI) are a significant source of patient morbidity and healthcare cost. Whether an SSI occurs depends upon a complex interaction between numerous factors, including the nature and number of organisms contaminating the surgical site, use of appropriate antimicrobial prophylaxis and other preventive measures, the health of the patient, and the technique of the surgeon. While many factors may contribute to an individual incidence, SSI rates have been shown to vary with wound closure method [1], with conflicting studies comparing sutures and staples. Older studies have suggested that tape-based skin closure is correlated with fewer infection-related complications than sutures, but until recently, no further comparisons have been published [2-4]. One study determined that tape-based wound closure produced wounds with higher tensile strength than sutures [5]. A new adjustable zip-type wound closure device (Zip Surgical Skin Closure, ZipLine Medical Inc., Campbell, CA, USA) has recently been introduced for closure of surgical wounds and lacerations. Our intent was to determine if this device, through its unique mechanism of wound closure, would enable less bacterial infiltration into a wound after closure, exposure to manipulation and contamination, than subcuticular sutures.

Wound closure for all but the most minor wounds traditionally consists of mechanical coupling of opposing wound edges by percutaneous or intradermal sutures or metal staples. With the exception of high-tension wounds, absorbable intradermal sutures are frequently used as they do not require subsequent removal. Metal staple wire and suture thread control tissue by creating a concentrated point-force or line-force of load bearing. Sutures and staples serve as intermittently direct means of closure, relying on tissue density or inelasticity to maintain closure between each discreet staple or suture pass. This can result in nonuniform compressive forces at the incision line [6]. The relatively high compressive forces incident on the local tissue from the wire or suture material has been shown to result in reduced blood flow and ischemia at the wound site [Poster: Davis A, Vaughn M, Piraino J. Effect of Surgical Incision Closure Device on Skin Perfusion Following Total Ankle Arthroplasty. Presented at American College of Foot and Ankle Surgeons; Feb 27-Mar 1, 2017; Las Vegas, NV. http://www.acfas.org/Regional-Divisions/Div-5-Davis-2017/. Last accessed 4/27/2018]. In addition, both staples and sutures by the very nature of their localized control of tissue at the incision line allow distraction forces to reach the incision. This mechanical force has been correlated to proliferation of scars, including the proliferation of keloids [7]. Early studies of non-invasive tape-based wound closure suggested less risk of surgical site infection, allegedly due to the absence of additional percutaneous punctures that can become pathways for bacteria to penetrate into the wound [8-11]. Unfortunately, these approaches had limited real-world applicability due to limited adhesive strength and adjustability to enable a desired wound closure tension. The zip-type device under study here has been demonstrated to prevent these forces from reaching the wound, with a resulting reduction in keloid and hypertrophic scar proliferation [12].

Normal patient movement can create distraction forces on the wound. An acute in vivo study comparing the mechanical forces of a zip-type wound closure to intradermal sutures suggested that the zip-type device resulted in a more uniform distribution of closure forces along the incision while making the incision less vulnerable to distraction forces [6]. Based on that mechanical analysis and result, we hypothesize that the zip-type device may also reduce bacterial penetration and resulting surgical site infection compared to intradermal sutures, in particular when the wound is subject to distraction forces that may be induced from patient movement during normal recovery.

An alternate form of closure that has the efficacy of sutures with a decreased risk of infection under real-world conditions (external contamination, distraction forces) has the potential to offer an improvement to the standard of care. This study compares normal absorbable subcuticular sutures to an adjustable polymer closure that is placed on the outside of the wound, the zip-type closure, and tests the bacterial burden and symptoms of different rates of infection in hairless guinea pigs in a post-surgical period where Staphylococcus aureus (S. aureus) is placed on the closed incision.

## Materials and methods

Wound closure devices

**Figure 1:**
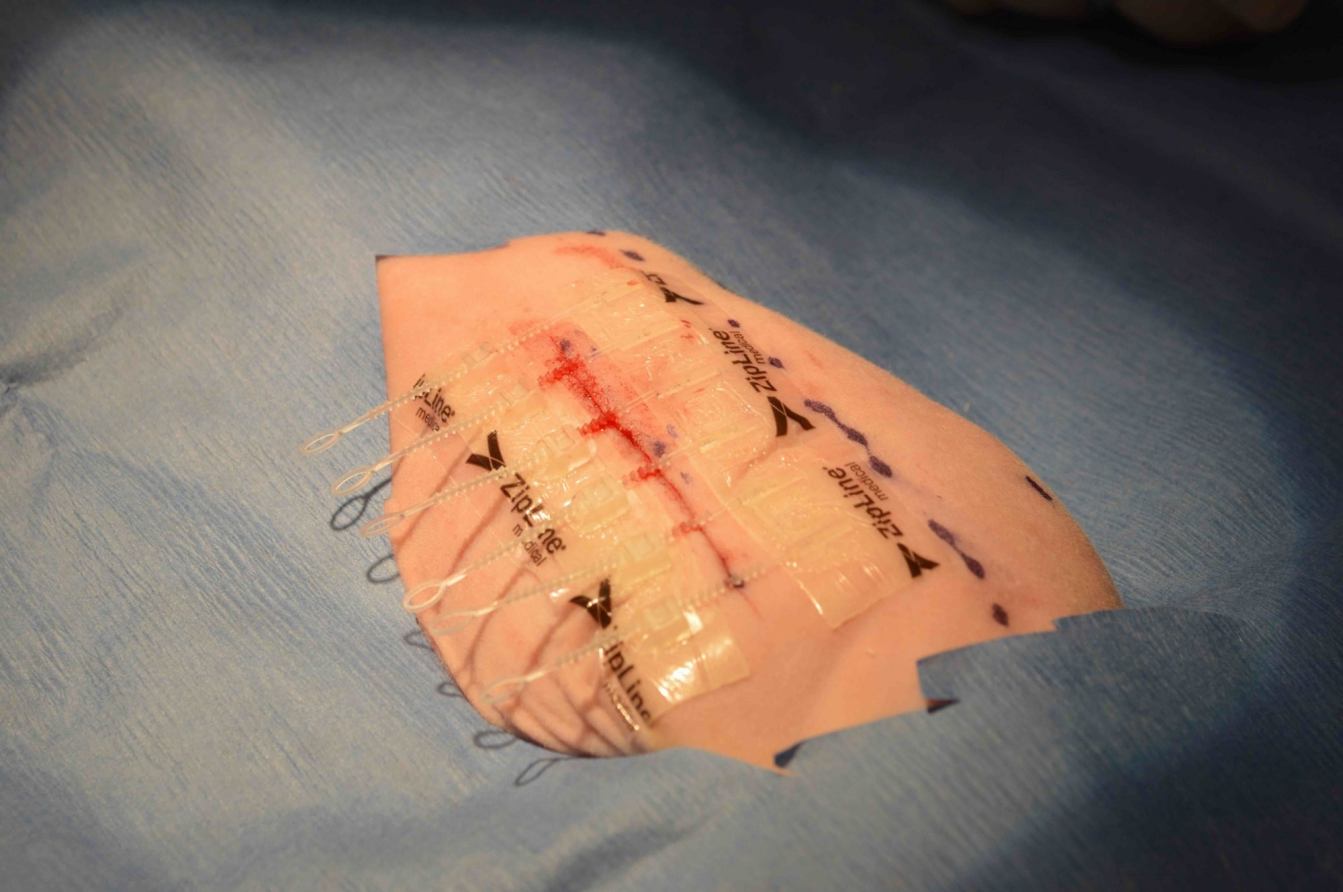
Zip closure device.

Absorbable sutures (4-0 Vicryl, Ethicon, Cincinnati, OH) and the Zip® 8i Surgical Skin Closure (ZipLine Medical, Inc., Campbell, CA) were used. All devices were provided sterile. The zip device (Figure 1) consists of two polyurethane strips attached to either side of a wound with a hydrocolloid pressure-sensitive skin adhesive. Closure is achieved by means of a series of interconnected nylon zip-tie-type ratcheting straps, with the terminus of each pair of locks and straps interconnected along each polyurethane strip with a force-distributing longitudinal nylon strut. The device enables adjustable closure at different force levels along the wound, and the longitudinal nylon struts distribute closing force from the straps to the adhesive strips between each strap. Levi et al. demonstrated that this apparatus provides greater shielding of the wound from perturbation caused by distraction forces than intradermal sutures [6].

Studies were performed on hairless male guinea pigs (Cavia porcellus) (n = 16) in accordance with institutional animal guidelines. General inhalational anesthesia and sedation were provided by veterinary staff. Animals were two to four months old and between 200 to 500 grams in weight. Guinea pigs were chosen because their skin shares many properties with human skin. A hairless variety was selected to prevent a guinea pig’s relatively rapid hair regrowth from interfering with study results.

**Figure 2:**
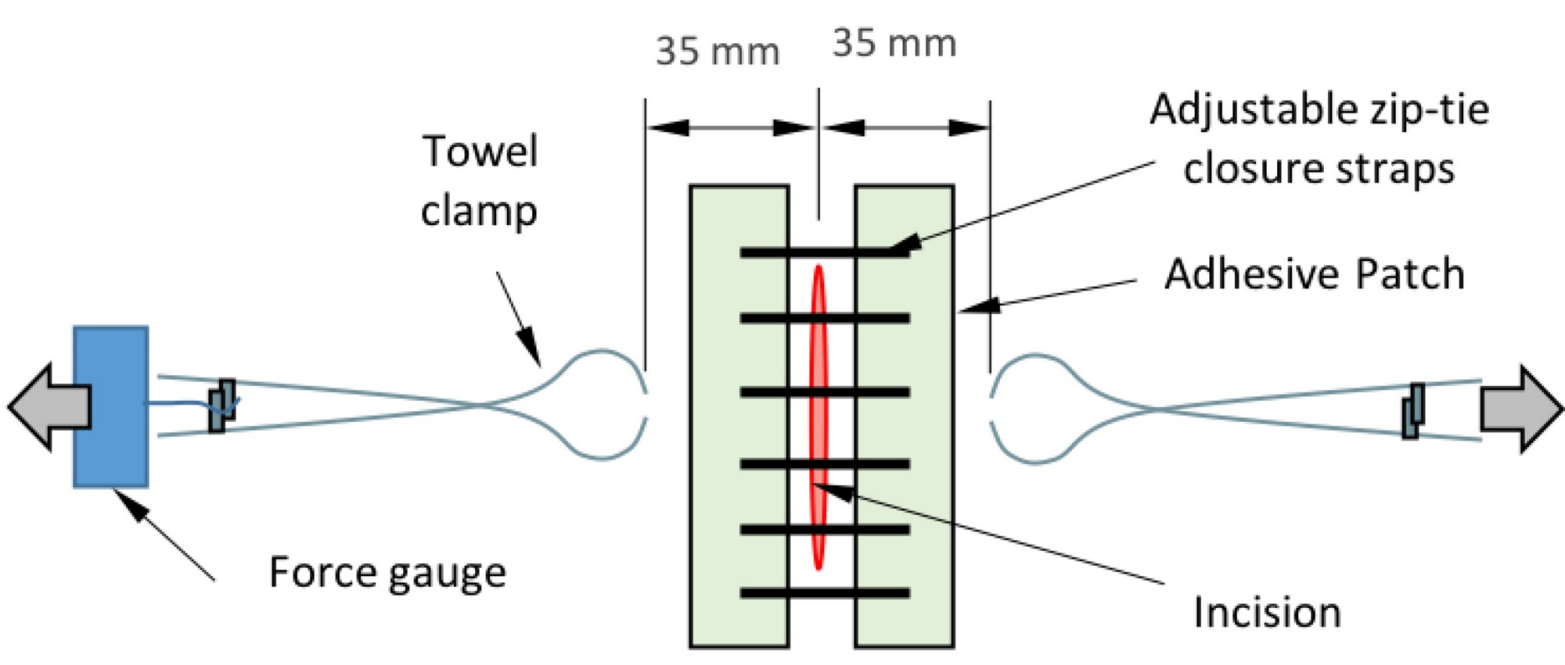
Distraction force apparatus.

On the day of the procedure, each guinea pig was maintained under anesthesia with isoflurane. The skin was swabbed with betadine, then wiped with a 70% alcohol soaked gauze. Incision creation and wound closure were performed by a board-licensed attending veterinarian. Two parallel 4 cm long full-thickness surgical incisions were created on each side of the pig dorsum. One incision was closed with the zip device and the other with a single intradermal running suture. After both incisions were closed, bacteria (S. aureus) was pipetted along the wound at 100 uL. A gentle distraction force of 0.75 pounds was then applied to each wound to simulate patient movement. This amount of force was selected based on proprietary testing of measured forces incurred in locations of surgical wounds from typical recovering patient movement, measured during product development. Towel clips were attached to the skin approximately 35 mm away from the incision (Figure 2). One towel clip was connected to a calibrated Chatillon Force Measurement Gauge, Model DFS2-025 (Ametek, Berwyn, PA) to measure force. Force was applied and released for a total of four cycles per wound. No bandages or tape were used to cover the incisions. For pain management, the animals were administered buprenorphine for two days post-surgery.

Photographs were taken from one to seven days post-surgery and clinical observations were made from the day of the surgery to the seventh day post-surgery. Body weight was taken for each guinea pig once a week. On the seventh day post-surgery, the guinea pigs were euthanized with ketamine/xylazine (IM) and euthasol (intracardiac) injections.

Bacterial quantification

Skin was excised seven days post-surgery and a 2 cm x 0.5 cm piece was minced, cultured and analyzed. The result was then assessed pair-wise, zip device versus suture closure, and the colony-forming units (CFU) of each compared. The mean, standard deviation (SD), and standard error of the mean (SEM) were all quantified with both Microsoft Excel 2013 (Microsoft Corporation, Redmond, Washington, USA) and Prism Graphpad (GraphPad Software, La Jolla, CA, USA). No statistical test for significance was performed.

Histology

Hematoxylin and eosin (H&E) stained skin sections taken at post-op day seven were evaluated for inflammation and the presence of bacterial infection. The tissues were examined by a veterinary pathologist certified by the American College of Veterinary Pathologists and scored on the industry standard five-point scoring system [13]. A score of zero indicated normal tissue, with only expected deviations from normal by the conditions of the study. A score of one indicated minimal inflammation with possible new collagen; the epidermis remains intact. A score of two indicated visible inflammation of limited severity. No functional impairment exists, but nonsuppurative inflammation is scattered, as is new collagen; the epidermis is still intact. A score of three indicated a lesion of moderate severity that has a potential to become increasingly severe. There is limited tissue or organ dysfunction. There may be abscesses or pustules with exudate, suture, and large numbers of bacteria. A score of four indicated extensive and severe inflammation with significant tissue or organ dysfunction. Large, ulcerating abscesses are present, with exudate, suture, and large numbers of bacteria.

## Results

All incisions in the test animals were fully healed by the end of the week. In all but one of the sample animals, weight gain was noted over the seven days post-surgery. The lone animal with weight loss was 2.7 grams lighter than the starting 100 grams weight; the greatest gain for any of the test animals was 12.7 grams. The zip devices started to partially lose adhesion as soon as the first post-surgical day; the first ones to completely detach began at the fourth day post-surgery. All had become detached by the seventh day. In all test animals, the wounds, whether sealed by suture or by zip device, had scabbed over by the end of the test period. There was no mortality of any of the test subjects in this period of time.

**Figure 3:**
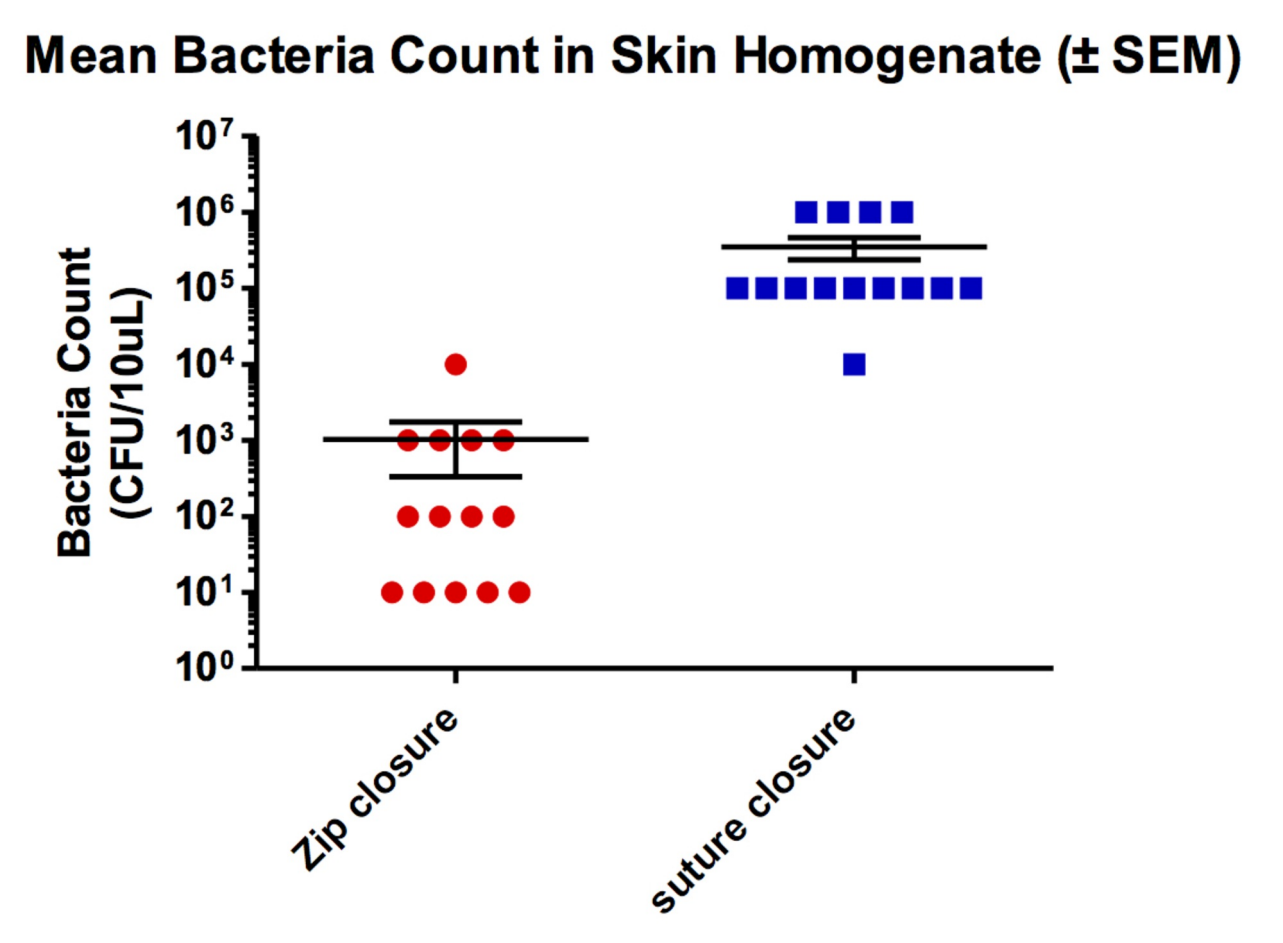
Comparison of suture vs. zip-type device for amount of bacteria in skin homogenate. SEM: Standard error of the mean.

All skin homogenate samples showed fewer bacteria in incisions closed with the zip device versus the sutured closure. Most notably, during the mincing procedure, pockets of oozing pus could be seen in the sutured wounds, but no pockets of pus could be observed in the incisions closed with the zip device. The mean bacteria count in the samples from the zip-closed incisions was 10^3^ CFU/10 uL, while the mean bacteria count in the sutured samples was 10^5^ CFU/10 uL (Figure 3). The percentage of difference in the bacteria noted in the samples was no less than 99% in all 14 and was 100% in 10 out of the 14.

Histology samples

**Figure 4:**
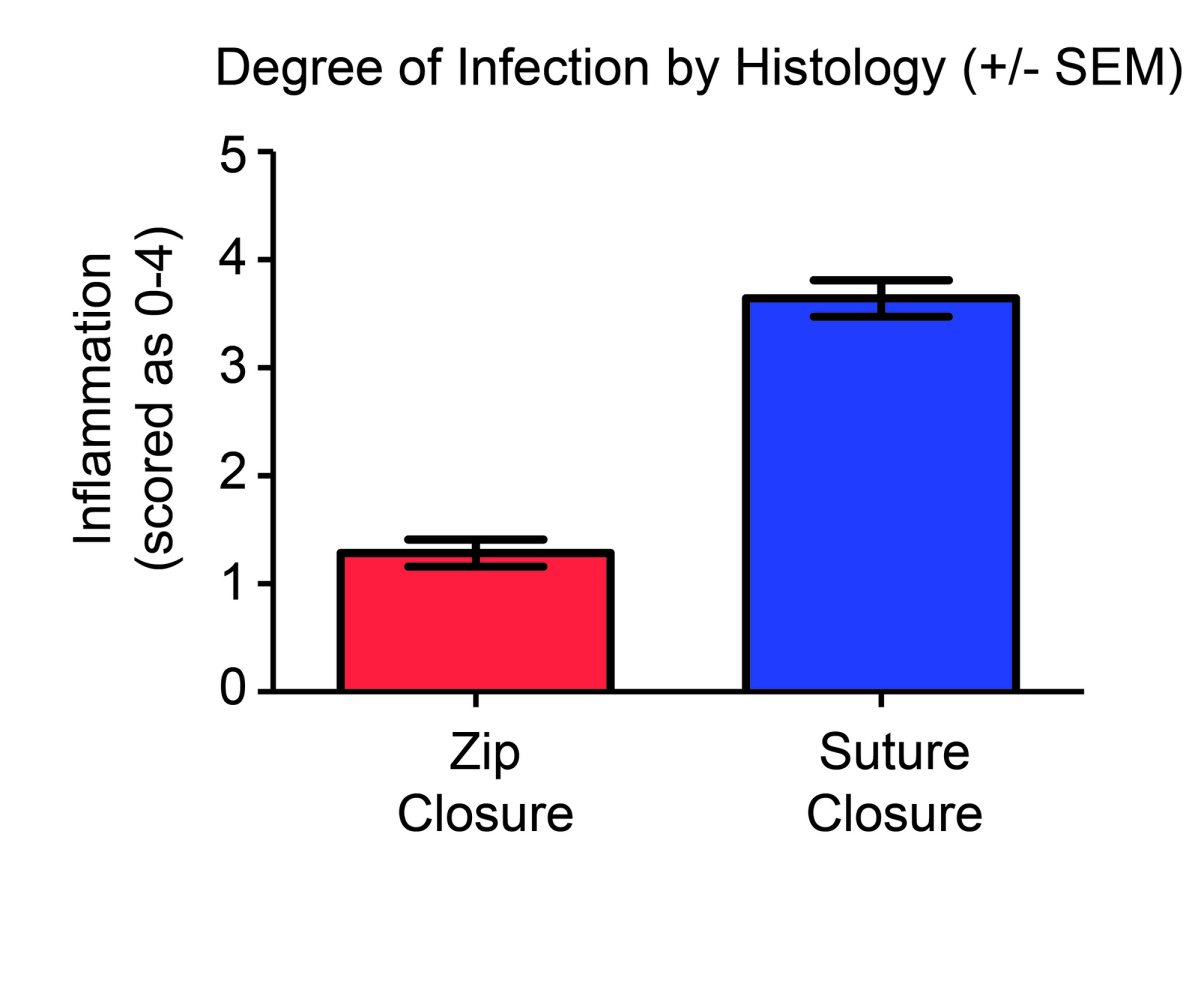
Degree of infection by histological analysis of H&E stained slides. 0 = within normal limits 1 = minimal 2 = slight 3 = moderate 4 = severe SEM: Standard error of the mean; H&E: Hematoxylin and eosin.

The histological samples were graded on a score of zero to four. The mean score for the samples from the zip-closed incisions was one, while the mean score for the sutured samples was three (Figure 4). The highest score present in the samples from the zip-closed incisions was a score of two; the lowest was a score of one. The sutured samples ranged from a score of two in one test animal to a score of four in 10 of the test animals.

**Figure 5:**
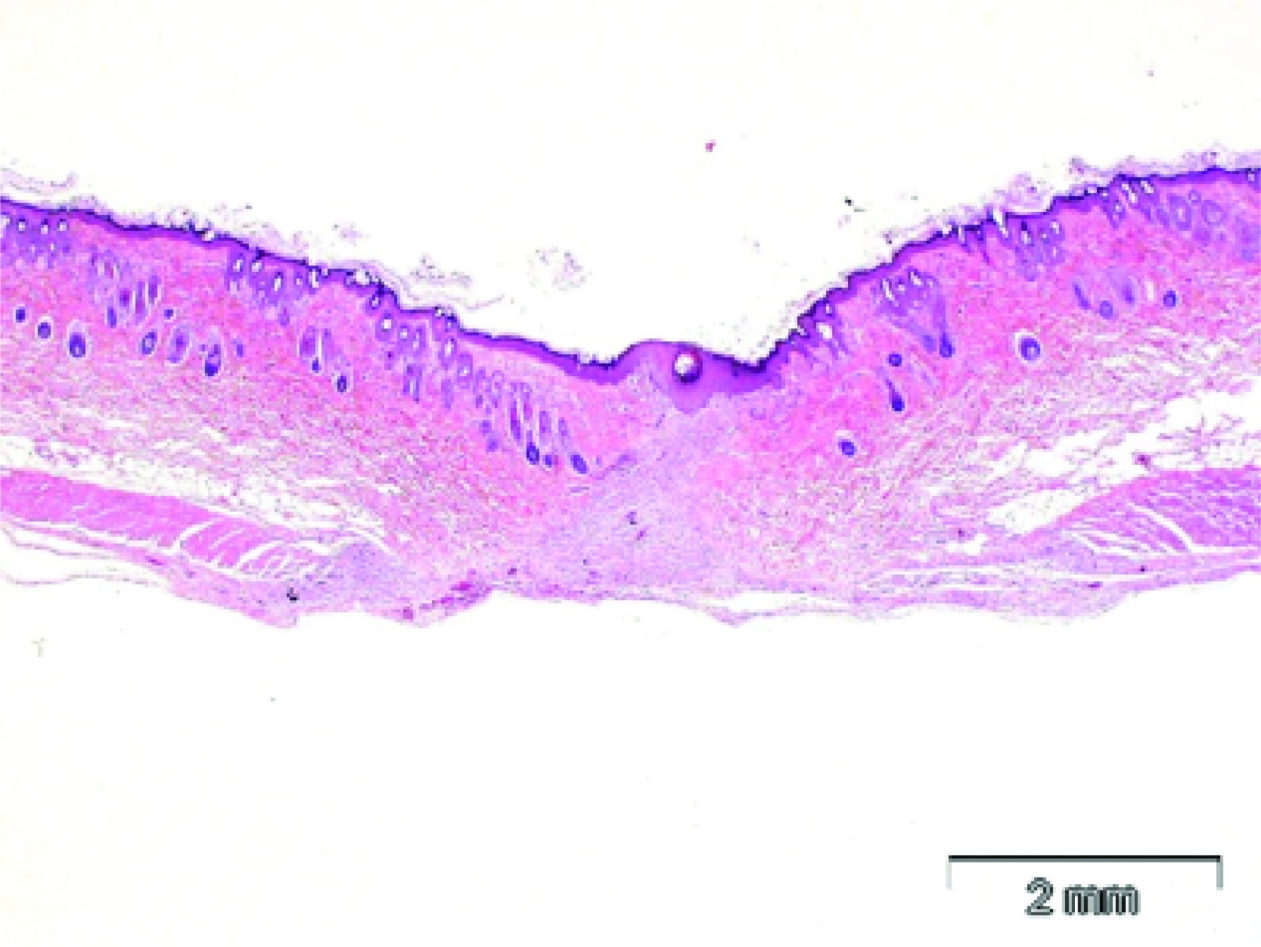
Skin, zip closure plus bacterial infection induction. In this full thick section of skin, the fully healed incision is visible in the center of the section. The surface is covered with a continuous and intact epidermis and the defect is filled with immature collagen. There is minimal inflammation, and no indication of bacterial infection or foreign body reaction. These findings indicate that the incision is completely healed and that no infection is present. H&E stain.

The epidermis was intact in all samples from the incisions closed with the zip device, and only residual inflammation with some new collagen formation could be found (Figure 5).

**Figure 6:**
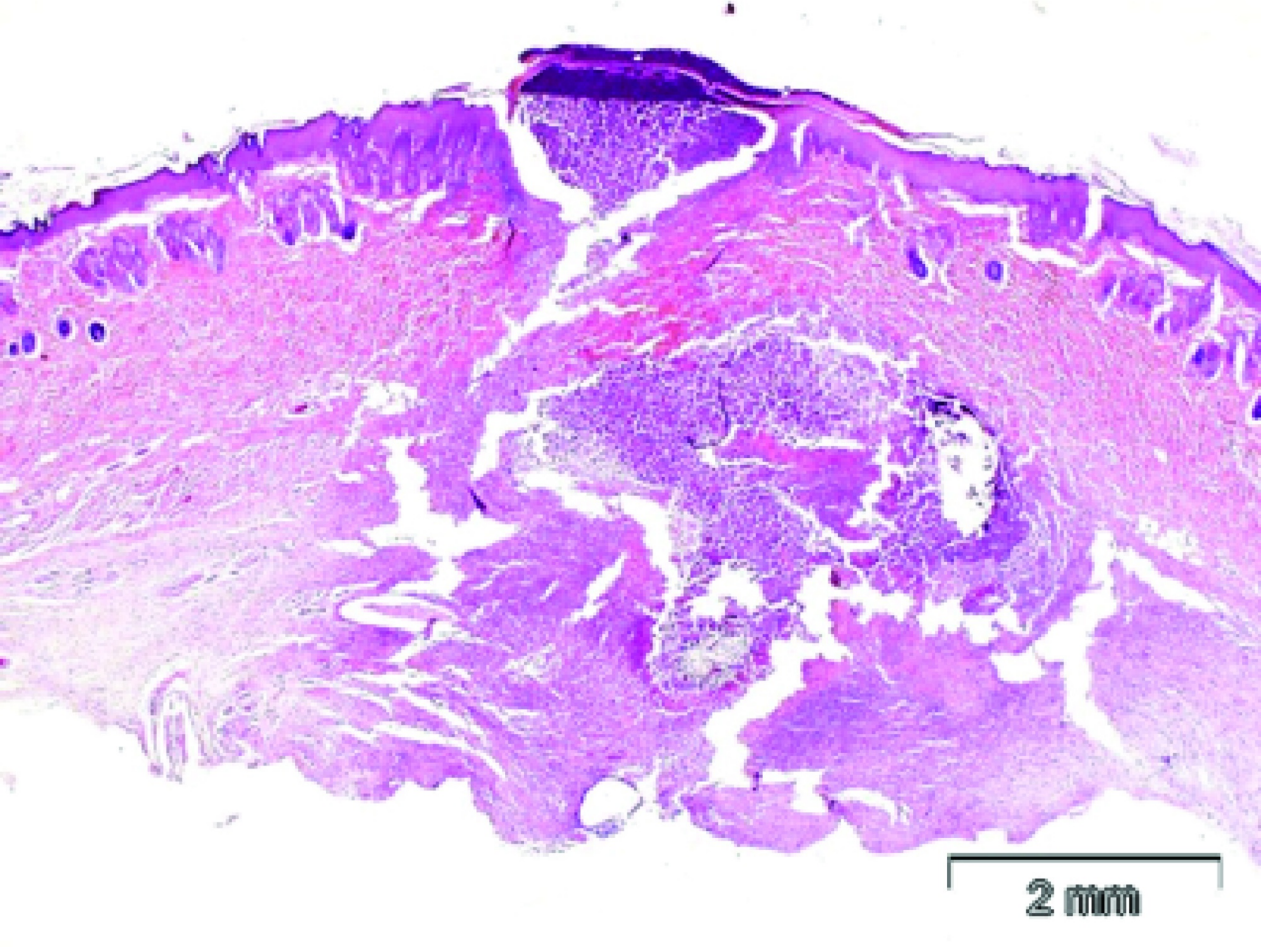
Skin, suture closure plus bacterial infection induction. In this full thickness section of skin, the incision is not healed and there is inflammation and a deep abscess extending into the deep center of the section. The epidermal surface at the incision site is discontinuous and covered with crust and purulent debris, and the adjacent epidermis is thickened and hyperplastic. In the deeper dermis, there is a large area of purulent inflammation with sutures and bacterial colonies present. These findings indicate that the incision is not healed and that infection is present. H&E stain.

**Figure 7:**
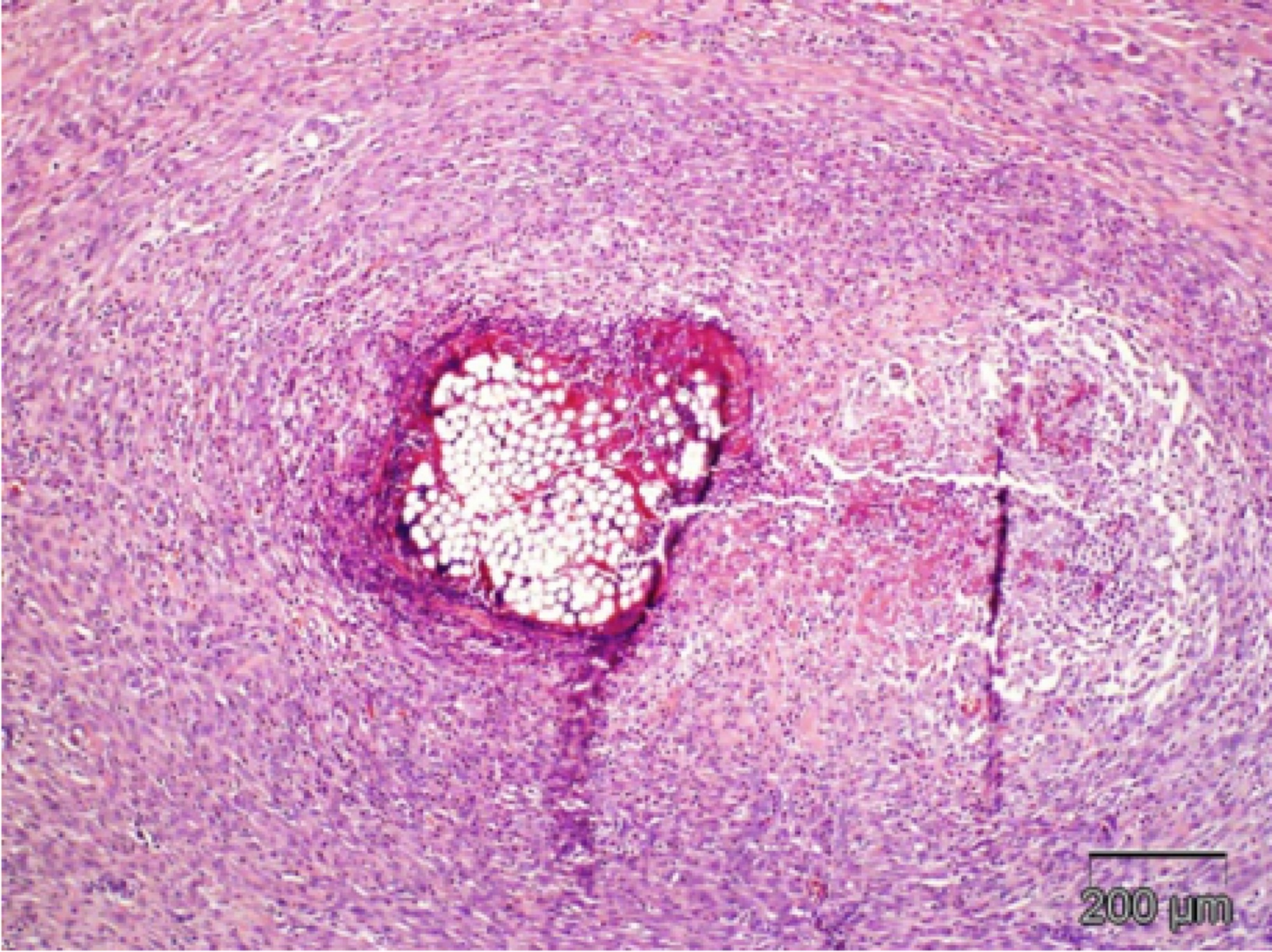
Skin, suture closure plus bacterial infection induction. In this high magnification photomicrograph of the deep dermis, there is intense chronic suppurative and foreign body inflammation surrounding a cross section of a suture. There are bacterial colonies associated with the suture. These findings indicate that the incision is not healed and that infection is present. H&E stain.

In contrast the sutured samples all had large intradermal abscesses, with suture material inside them (Figures 6-7). There were areas of purulent exudate containing pus, neutrophils, debris, and large numbers of bacteria. Lack of inflammation in the samples from the incisions closed by the zip device may indicate that formation of scar tissue would be lower.

## Discussion

This study had a number of limitations. First, although quantified, a gentle pulling on the incision represented an arbitrary level of force at a single point in time immediately after wound closure. Distraction force caused by patient movement may occur at any time and at varying degrees post-operatively. A single bacterial strain (S. aureus) was used as this represents a common cause of SSI. The bacteria was intentionally pipetted onto the closed wounds in an attempt to amplify any relative differences between the two closure methods to produce a detectable signal with such a small sample size. This study was not powered to measure the rate of surgical site infection nor was that a primary aim due to a lack of preliminary data or literature precedent for test methodology or expected value of bacterial penetration.

## Conclusions

The initial data in this study indicate less bacteria in the wound site and a possible lower rate of infection in surgical incisions closed with the adjustable tape-based zip device compared to incisions closed with subcuticular sutures. From the lack of inflammation in incisions closed with the zip device, it may be possible that the closure will result in less scar formation than in incisions closed with subcuticular sutures. Further studies with human subjects are warranted.
